# Nonlinear relationship between visceral adiposity index and lung function: a population-based study

**DOI:** 10.1186/s12931-021-01751-7

**Published:** 2021-05-24

**Authors:** Yide Wang, Zheng Li, Fengsen Li

**Affiliations:** 1 Department of Integrated Pulmonology, Fourth Clinical Medical College of Xinjiang Medical University, 116 Huanghe Road, Urumqi, Xinjiang China; 2 Xinjiang National Clinical Research Base of Traditional Chinese Medicine, Xinjiang Medical University, 116 Huanghe Road, Urumqi, Xinjiang China

**Keywords:** Obesity, Visceral adiposity index, Lung function, Cross-sectional studies

## Abstract

**Background:**

As one of the critical indicators of obesity, the interaction between visceral fat content and lung disease is the focus of current research. However, the exact relationship between Visceral adipose index (VAI) and lung function is not fully understood. The purpose of this study was to evaluate the relationship between VAI and lung function,

**Methods:**

Our study included all participants from the baseline survey population in Xinjiang in the Natural Population Cohort Study in Northwest China. A field survey was conducted in rural areas of Moyu County, Xinjiang, China, between 35 and 74 years old from June to December 2018. We collected standard questionnaires and completed physical examinations, visceral fat tests, and lung function measurements.

**Results:**

The study included 2367 participants with a mean VAI of 10.35 ± 4.35, with males having a significantly higher VAI than females: 13.17 ± 3.91 vs. 7.58 ± 2.65. The piecewise linear spline models indicated a significant threshold effect between lung function and VAI in the general population and the males population, showing an inverted U-shaped curve. But there was no significant association between VAI and lung function in females. FEV1% predicted and FVC% predicted increased with the increase of VAI (β 0.76; 95% CI 0.30, 1.21) and (β 0.50; 95% CI 0.06, 0.94) in males with VAI ≤ 14, while FEV1% predicted and FVC% predicted decreased with the increase of VAI (β − 1.17; 95% CI − 1.90, − 0.45) and (β − 1.36; 95% CI − 2.08, − 0.64) in males with VAI ≥ 15.

**Conclusions:**

The relationship between lung function and VAI in male participants showed an inverted U-shaped curve, with the turning point of VAI between 14 and 15. The association between visceral fat and lung function was more robust in males than in females.

**Supplementary Information:**

The online version contains supplementary material available at 10.1186/s12931-021-01751-7.

## Background

Obesity is a chronic metabolic disease with excessive accumulation or abnormal distribution of total or local fat content in the body [1]. Over the past 40 years, the incidence of obesity has been on the rise worldwide [2, 3]. As a complex multi-factor disease, obesity has caused more and more heavy social and economic burden. Obesity significantly increases susceptibility to respiratory disease than healthy controls. It is one of the critical factors contributing to respiratory diseases such as chronic obstructive pulmonary disease, asthma and pulmonary hypertension [4–6]. Obesity has an essential effect on lung function. Most studies believe that obesity-related indicators are negatively correlated with lung function changes [7, 8].

Abdominal obesity is a significant risk factor for lung function impairment and metabolic syndrome [9]. A study has found that visceral fat plays a significant role in lung function damage caused by abdominal obesity [10]. Clinical measurements such as body mass index (BMI) and waist circumference (WC) provide minimal information on fat distribution, and there is no way to distinguish between subcutaneous and visceral fat. Visceral adipose index (VAI) is a good indicator of visceral fat function and distribution. VAI is closely related to metabolic syndrome, diabetes and other diseases [11, 12], but the specific relationship between VAI and lung function is still unclear.

Most of the studies that have been done to correlate with impaired lung function have been linear studies with increases or decreases [13–16]. However, in real-world biomedical research, the influence of many research factors on outcome variables is not a simple linear relationship but exists the phenomenon of a threshold effect. There is a positive or negative effect within a certain range, but the direction of development changes after passing a certain cut-off point [17]. This study tried to use the smoothing curve fitting method to analyze the specific relationship between VAI and lung function impairment and tried to find the possible cut-off point.

## Methods

### Study population

This study is a branch of the “Natural Population Cohort Study in Northwest China” project. All data were collected from a baseline survey in rural areas of Moyu County, Northwest China, between June and December 2018. Participants were excluded if one of the following criteria were met: (1) Be under 35 years old; (2) Lack of essential information such as gender, age and education level; (3) People who have not completed lung function and visceral fat tests. In the end, a total of 2367 people were included in the study, including 1171 males and 1196 females. The Ethics Committee of Xinjiang Uygur Autonomous Region Chinese Medical Hospital approved the study protocol, and informed consent was obtained from each participant. Our study also complied with the Declaration of Helsinki.

### Data collection

We used standardized questionnaires to obtain demographic characteristics (such as age, sex, education, etc.), smoking status, and the participants' medical history. The staff consists of medical background and full-time investigators, who receive systematic training and field practice before the investigation. After being qualified, the investigators conducted face-to-face questionnaire surveys on the participants at each survey site's administrative village health service centre. The questionnaire was developed by the Natural Population Cohort Study Project Group in Northwest China.

When measuring the height, take off the shoes and cap, and use the medical height meter to measure (the accuracy is 0.1 cm); When measuring the weight, take off the coat, shoes and cap, and use the electronic medical scale to measure (accuracy is 0.1 kg); BMI = weight (kg)/ height (m)^2^. VAI was determined by Tanita DC-430MA human body component analyzer (Tanita DC430MA, Tokyo, Japan). Before the test, the participants should try not to drink water, eat food, do strenuous exercise, and empty urine. The participants were asked to stand on the tester with bare feet, with the forefoot and back heel of both feet respectively on the foot electrode of the tester, hold the hand electrode with both hands, and contact the corresponding electrode points with the thumb, palm and the remaining four fingers respectively. The body relaxes, the upper limbs droop naturally, through the painless current, the body's resistance to the current is measured, and the result is obtained. Lung function was measured using a Masterscreen Pneumo (version2.13, Germany). Operate according to the American Thoracic Society/European Respiratory Society (ATS/ERS) guidelines [18]. All procedures required participants to sit, wear a nose clip, use a disposable mouthpiece, and repeat the measurement to ensure accuracy.

### Statistical analysis

Continuous variables such as age, BMI and so on are represented by means ± standard deviations. In contrast, classification variables such as gender, education level, smoking status and so on are represented by numbers (percentages). The basic characteristics of the participants were described according to different genders. Unpaired Student's t-test was used for continuous variables comparison, and the Pearson chi-square test was performed on classified variables. The characteristics of people with different VAI levels were analyzed. One-way analysis of variance was used for continuous variables.

We took VAI as the predictor variables and lung function as the response variables to construct a smoothing fitting curve. The threshold level (turning point) for each was determined using likelihood-ratio tests and bootstrap resampling methods. Further, We tested the specific relationship between VAI and lung function using a piecewise regression model. Confounding variables include age, education level, gross annual income, marital status, active smoking, passive smoking, hypertension, COPD, Chronic bronchitis, osteoporosis, fracture, peptic ulcer, cholecystitis, and CKD. Besides, possible modifications on the relation of VAI levels with FEV1% predicted and FVC% predicted were evaluated by stratified analyses. All variables including age (< 65 vs. ≥ 65 years), Education level (illiterate vs. primary and so on), Gross annual income (< 10,000 vs. ≥ 10,000 yuan), Marital status (married vs. Divorce and so on), Active smoking (Yes vs. No), Passive smoking (Yes vs. No) and more, did not significantly modify the association between VAI and Lung function.

Data analyses were carried out using STATA version 14.0 (STATA Corp., College Station, Texas), and Empower(R) (www.empowerstats.com, X&Y Solutions, Inc. Boston, MA). A *P* value of ≤ 0.05 was considered statistically significant.

## Results

### Study participants and characteristics

The basic characteristics of the participants were described by gender (Additional file 1: Table S1). A total of 2367 participants, aged 53.61 ± 9.46 years, were included in this study, including 1171 males and 1196 females. There were significant statistical differences in age, BMI, VAI, economic income, smoking and other factors between different genders. The mean VAI for all participants was 10.35 ± 4.35, with 13.17 ± 3.91 for males and 7.58 ± 2.65 for females. Compared with females, the FVC% predicted for male lung function was lower, 97.47 ± 19.38% and 95.63 ± 19.80%, respectively. Females’ FEV1% predicted were also slightly higher than males', 89.23 ± 20.18% and 86.79 ± 20.65, respectively. The active smoking rate of local males was significantly higher than that of local females (26.30% vs. 0.67%).

This study also analyzed differences in baseline characteristics among people with different VAI levels (Table 1). Participants with higher VAI levels were significantly associated with males, older age, higher income, higher prevalence of hypertension, lower prevalence of cholecystitis, and more. Participants with slightly higher VAI (10–14) and higher VAI (15–30) had higher pulmonary function FEV1%predicted than those with standard VAI (1–9). Participants with slightly higher VAI (10–14) had higher FVC% predicted than those with high VAI (15–30) and standard VAI (1–9) levels.

**Table 1:** Characteristics of 1196 females and 1171 males among 3 VAI categories in this study

Variables	VAI			P-value
	Q1 (1 ~ 9) n = 1123	Q2 (10 ~ 14) n = 776	Q3 (15 ~ 30) n = 468	
Sex*				
Male	183 (16.30%)	532 (68.56%)	456 (97.44%)	< 0.001
Female	940 (83.70%)	244 (31.44%)	12 (2.56%)	
Age, year^Δ^	51.48 ± 9.06	53.70 ± 9.18	58.57 ± 8.95	< 0.001
BMI, kg/m^2Δ^	23.69 ± 3.43	26.57 ± 4.20	29.12 ± 3.68	< 0.001
Marital status*				
Married	898 (79.96%)	672 (86.60%)	428 (91.45%)	< 0.001
Divorce and so on	225 (20.04%)	104 (13.40%)	40 (8.55%)	
Education level*				
Illiterate	166 (14.78%)	114 (14.69%)	69 (14.74%)	0.998
Primary and so on	957 (85.22%)	662 (85.31%)	399 (85.26%)	
Gross annual income, yuan*				
< 10,000	675 (60.11%)	400 (51.55%)	224 (47.86%)	< 0.001
≥ 10,000	448 (39.89%)	376 (48.45%)	244 (52.14%)	
Active smoking*				
Yes	73 (6.50%)	153 (19.72%)	90 (19.23%)	< 0.001
No	1050 (93.50%)	623 (80.28%)	378 (80.77%)	
Passive smoking*				
Yes	52 (4.63%)	78 (10.05%)	31 (6.62%)	< 0.001
No	1071 (95.37%)	698 (89.95%)	437 (93.38%)	
Chronic bronchitis*				
Yes	219 (19.50%)	130 (16.75%)	75 (16.03%)	0.152
No	904 (80.50%)	646 (83.25%)	393 (83.97%)	
COPD*				
Yes	138 (12.29%)	88 (11.34%)	64 (13.68%)	0.476
No	985 (87.71%)	688 (88.66%)	404 (86.32%)	
Hypertension*				
Yes	229 (20.39%)	224 (28.87%)	183 (39.10%)	< 0.001
No	894 (79.61%)	552 (71.13%)	285 (60.90%)	
Peptic ulcer*				
Yes	98 (8.73%)	54 (6.96%)	29 (6.20%)	0.152
No	1025 (91.27%)	722 (93.04%)	439 (93.80%)	
Cholecystitis*				
Yes	297 (26.45%)	146 (18.81%)	82 (17.52%)	< 0.001
No	826 (73.55%)	630 (81.19%)	386 (82.48%)	
CKD*				
Yes	68 (6.06%)	65 (8.38%)	39 (8.33%)	0.098
No	1055 (93.94%)	711 (91.62%)	429 (91.67%)	
Osteoporosis*				
Yes	84 (7.48%)	47 (6.06%)	27 (5.77%)	0.323
No	1039 (92.52%)	729 (93.94%)	441 (94.23%)	
Fracture*				
Yes	70 (6.23%)	52 (6.70%)	32 (6.84%)	0.874
No	1053 (93.77%)	724 (93.30%)	436 (93.16%)	
FVC% predicted^Δ^	95.80 ± 19.08	97.60 ± 19.52	96.53 ± 20.91	0.151
FEV1%predicted^Δ^	86.49 ± 20.07	89.36 ± 20.48	89.37 ± 21.09	0.003

*BMI* body mass index, *VAI* visceral adiposity index, *CKD* chronic kidney disease, *FEV1* forced expiratory volume in 1 s, *FVC* forced vital capacity

*Categorical variables are presented as frequency and percentage, n (%), using Pearson chi-square test to compare differences

^Δ^Continuous variables are presented as mean value ± standard deviation, and one-way analysis of variance is used to compare the differences between each group

### Association of the VAI with impaired lung function

**Fig. 1:**
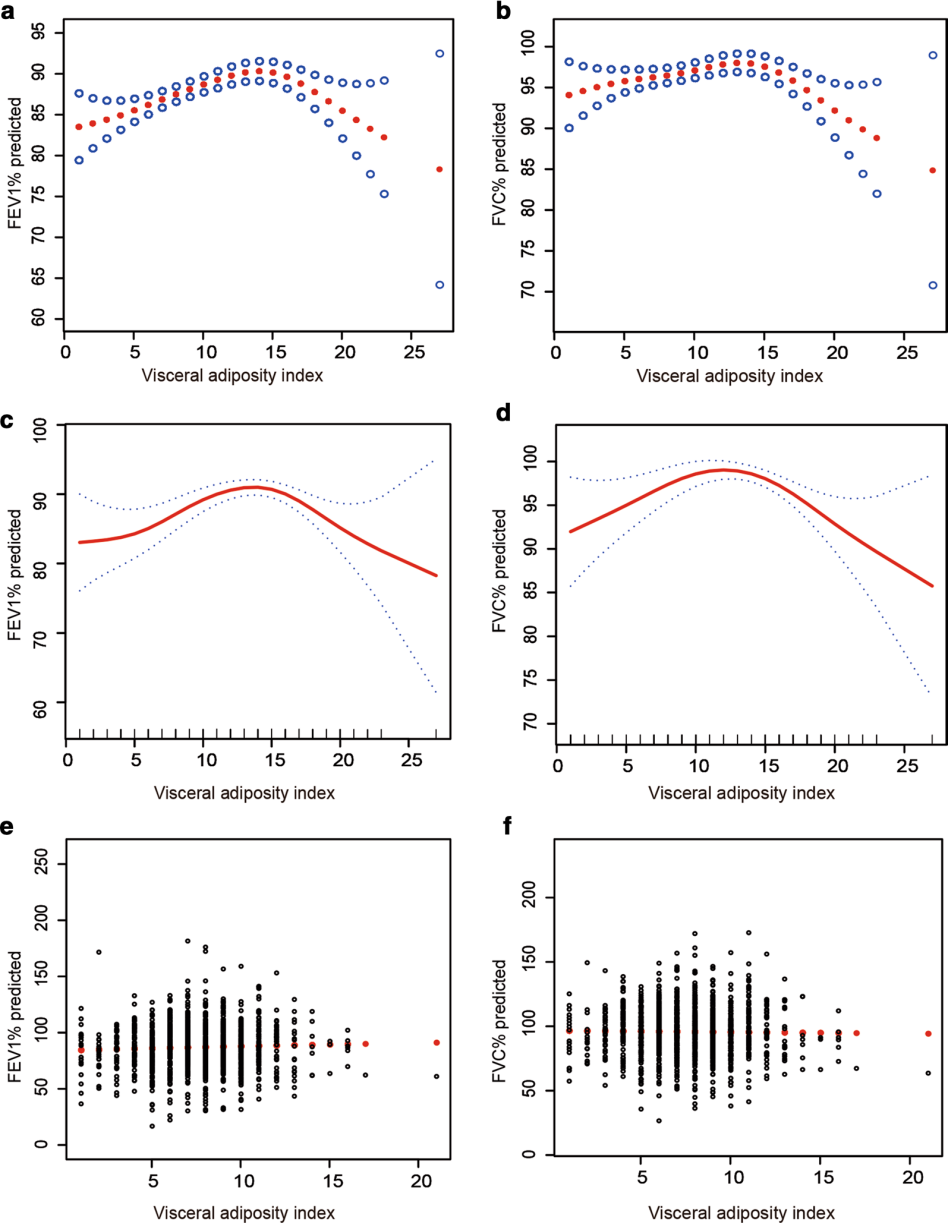
Association analysis between VAI level and lung function. Relationship of VAI levels with lung function FEV1% predicted (**a**) and FVC% predicted (**b**) in all the participants. Relationship of VAI levels with lung function FEV1% predicted (**c**), and FVC% predicted (**d**) in male participants. Relationship of VAI levels with lung function FEV1% predicted (**e**), and FVC% predicted (**f**) in female participants. Adjusted for age, economic income, education level, smoke exposure and other vital covariates including previous chronic bronchitis, COPD, hypertension, etc. *FEV1* forced expiratory volume in 1 s, *FVC* forced vital capacity, *VAI* visceral adiposity index, *COPD* Chronic obstructive pulmonary disease

The relationship between VAI and FEV1% predicted and FVC% predicted showed an inverted U-shaped curve (Fig. 1a, b). For FEV1% predicted, a cut-off point of VAI = 14.9 yielded the best fitting model in a piecewise regression, after fully adjusting for Age, economic income, education level, smoke exposure and other vital covariates including previous chronic bronchitis, COPD, hypertension, etc. one unit increase of VAI was significantly associated with 0.61 (95% CI 0.38,0.85) FEV1%predicted increase when VAI < 14.9, and one unit increase of VAI was significantly associated with − 1.70 (95% CI (− 2.63, − 0.77) FEV1%predicted decrease when VAI ≥ 14.9. For FVC% predicted, a turning point of VAI = 14.7 yielded the best fitting model in a piecewise regression, after fully adjusting for Age, economic income, education level, smoke exposure and other vital covariates including previous chronic bronchitis, COPD, hypertension, etc. one unit increase of VAI was significantly associated with 0.40 (95% CI 0.15,0.62) FVC% predicted increase when VAI < 14.7, and one unit increase of VAI was significantly associated with − 1.61 (95% CI (− 2.50, − 0.81) FVC% predicted decrease when VAI ≥ 14.7 (Table 2).

**Table 2:** Threshold effect analyses of association between VAI levels and lung function FEV1% predicted, FVC% predicted using two-piecewise regression models in all the participants

VAI	^*^Unadjusted	P-value	VAI	^Δ^Adjusted	P-value
	β(95%CI)			β(95%CI)	
FEV1% predicted					
< 15.0	0.63 (0.39, 0.87)	< 0.001	< 14.9	0.61 (0.38, 0.85)	< 0.001
≥ 15.0	− 1.54 (− 2.40, − 0.68)	< 0.001	≥ 14.9	− 1.70 (− 2.63, − 0.77)	< 0.001
Likelihood ratio test p		< 0.001			< 0.001
FVC% predicted					
< 15.0	0.37 (0.13, 0.60)	0.002	< 14.7	0.40 (0.15, 0.62)	0.002
≥ 15.0	− 1.60 (− 2.43, − 0.77)	< 0.001	≥ 14.7	− 1.61 (− 2.50, − 0.81)	< 0.001
Likelihood ratio test p		< 0.001			< 0.001

*VAI* visceral adiposity index, *FEV1* forced expiratory volume in 1 s, *FVC* forced vital capacity

*No variables have been adjusted

^Δ^Adjusted for Age, economic income, education level, smoke exposure and other vital covariates including previous chronic bronchitis, COPD, hypertension

Similarly, in male participants, the relationship between VAI and FEV1% predicted and FVC% predicted also showed an inverted U-shaped curve (Fig. 1c, d). For FEV1% predicted, a cut-off point of VAI = 14.6 yielded the best fitting model in an adjusted piecewise regression. One unit increase of VAI was significantly associated with 0.76 (95% CI 0.30, 1.21) FEV1%predicted increase when VAI < 14.6 in males, and one unit increase of VAI was significantly associated with -1.17 (95% CI − 1.90, − 0.45) FEV1%predicted decrease when VAI ≥ 14.6. For FVC% predicted, a cut-off point of VAI = 14.3 yielded the best fitting model in an adjusted piecewise regression. One unit increase of VAI was significantly associated with 0.50 (95%CI: 0.06, 0.94) FVC% predicted increase when VAI < 14.3 in males, and one unit increase of VAI was significantly associated with − 1.36 (95% CI − 2.08, − 0.64) FVC% predicted decrease when VAI ≥ 14.3 (Table 3).

**Table 3:** Threshold effect analyses of association between VAI levels and lung function FEV1% predicted、FVC% predicted using two-piecewise regression models in male participants

VAI	*Unadjusted	P-value	VAI	^Δ^Adjusted	P-value
	Β (95% CI)			Β (95% CI)	
FEV1% predicted					
< 14.0	0.78 (0.33, 1.23)	< 0.001	< 14.6	0.76 (0.30, 1.21)	0.001
≥ 14.0	− 1.14 (− 1.86, − 0.42)	0.002	≥ 14.6	− 1.17 (− 1.90, − 0.45)	0.002
Likelihood ratio test p		< 0.001			< 0.001
FVC% predicted					
< 14.1	0.50 (0.07, 0.93)	0.022	< 14.3	0.50 (0.06, 0.94)	0.025
≥ 14.1	− 1.38 (− 2.09, − 0.66)	< 0.001	≥ 14.3	1.36 (− 2.08, − 0.64)	< 0.001
Likelihood ratio test p		< 0.001			< 0.001

*VAI* visceral adiposity index, *FEV1* forced expiratory volume in 1 s, *FVC* forced vital capacity

*No variables have been adjusted

^Δ^Adjusted for Age, economic income, education level, smoke exposure and other vital covariates including previous chronic bronchitis, COPD, hypertension

However, as shown in Fig. 1e, f, and Additional file 2: Table S2, there was no significant association between VAI and FEV1% predicted or FVC% predicted in females.

### Stratified analyses by important covariables

**Fig. 2:**
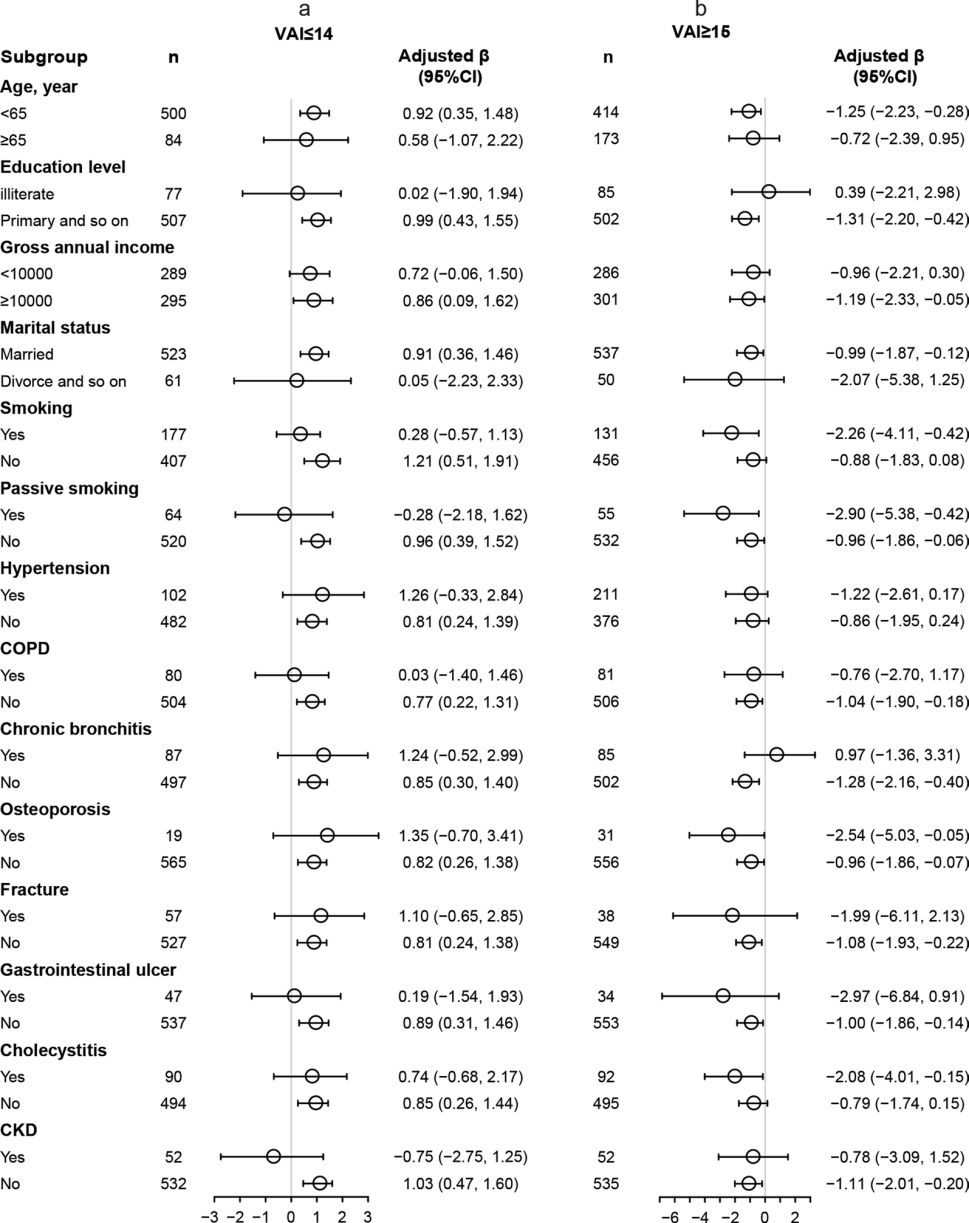
Stratified analysis for Relationship of VAI levels with FEV1% predicted in various subgroups divided at 14 (**a** VAI ≤ 14, **b** VAI ≥ 15) in male participants. Each subgroup analysis adjusted for age, education level, gross annual income, marital status, active smoking, passive smoking, hypertension, COPD, chronic bronchitis, osteoporosis, fracture, peptic ulcer, cholecystitis, and CKD. *FEV1* forced expiratory volume in 1 s, *VAI* visceral adiposity index, *COPD* Chronic obstructive pulmonary disease, *CKD* chronic kidney disease

We further performed exploratory subgroup analyses to assess the association between VAI and FEV1%predicted in two groups of participants divided at the turning point of VAI = 14.6. All variables including age (< 65 vs. ≥ 65 years), Education level (illiterate vs. primary and so on), Gross annual income (< 10,000 vs. ≥ 10,000 yuan), Marital status (married vs. Divorce and so on), Active smoking (Yes vs. No), Passive smoking (Yes vs. No), Hypertension (Yes vs. No), COPD (Yes vs. No), Chronic bronchitis (Yes vs. No), Osteoporosis (Yes vs. No), Fracture (Yes vs. No), Peptic ulcer (Yes vs. No), Cholecystitis (Yes vs. No), and CKD (Yes vs. No) alone, did not significantly modify the association between VAI and FEV1%predicted (Fig. 2).

**Fig. 3:**
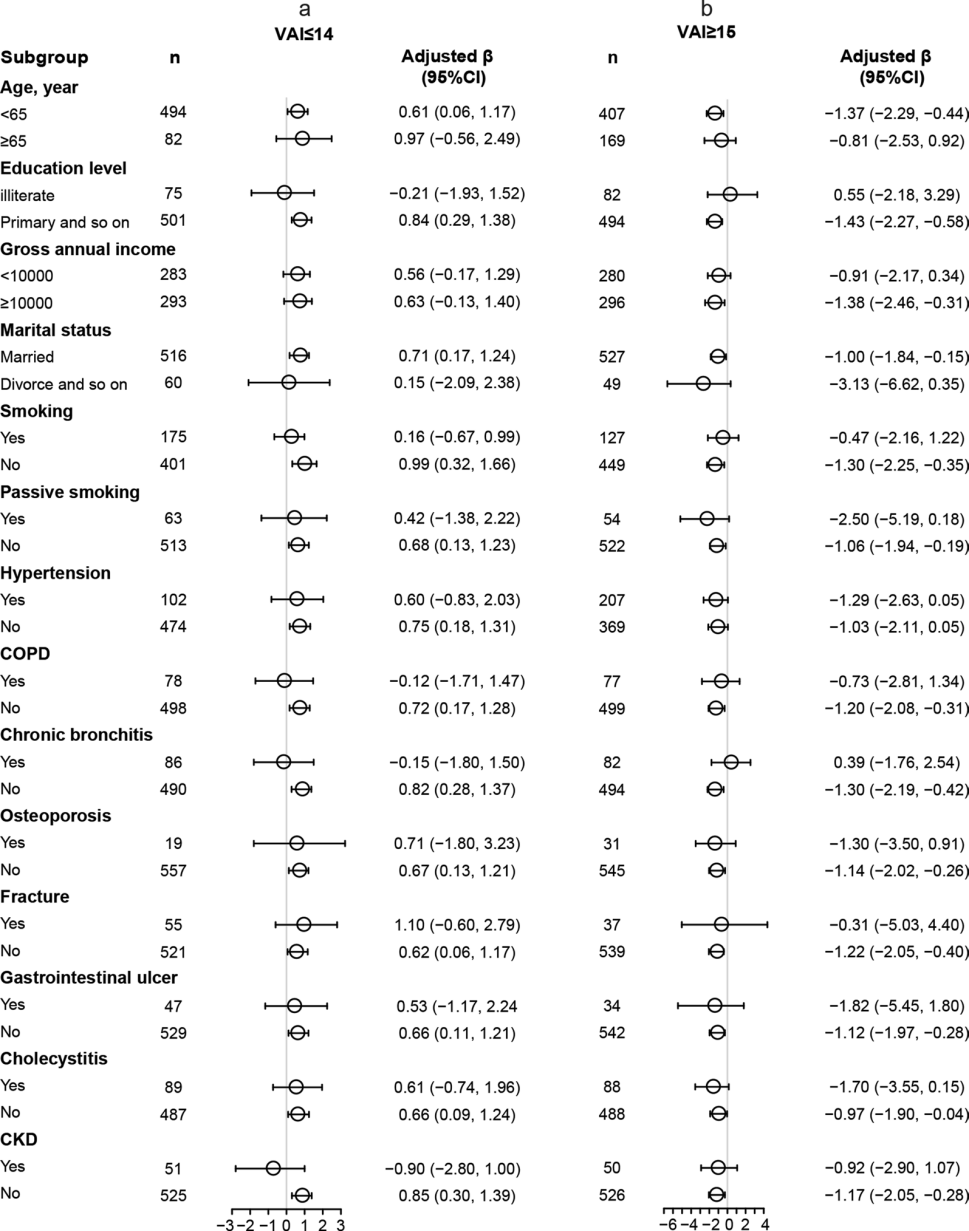
Stratified analysis for Relationship of VAI levels with FVC% predicted in various subgroups divided at 14 (**a** VAI ≤ 14, **b** VAI ≥ 15) in male participants. Each subgroup analysis adjusted for age, Education level, Gross annual income, Marital status, Active smoking, Passive smoking, Hypertension, COPD, Chronic bronchitis, Osteoporosis, Fracture, Peptic ulcer, Cholecystitis, and CKD. *FVC* forced vital capacity, *VAI* visceral adiposity index, *COPD* Chronic obstructive pulmonary disease, *CKD* chronic kidney disease

Similarly, after stratified analysis of the above variables, the relationship between VAI and FVC% predicted did not change significantly (Fig. 3).

## Discussion

In this study, the relationship between VAI and lung function in the local population was analyzed based on the Xinjiang rural areas’ baseline data in the Natural Population Cohort Study in Northwest China. To our knowledge, this is the first study to find an inverted U-shaped relationship between VAI and FEV1%predicted, FVC% predicted in a Chinese male population, and to identify a specific range of turning points: between 14 and 15 for VAI. This relationship persisted even after adjusting for confounders utilizing stratified analysis. However, no significant association was found between VAI and lung function in female. These findings remind us of the need for early lung function assessment in local populations with too low or too high VAI levels, especially in males.

A study using Asian-Pacific BMI classifications studied the relationship between obesity and lung function, and the results showed that BMI was negatively correlated with lung function [19]. However, FEV and the lung's diffusing capacity for carbon monoxide percentage revealed an inverse "U"-shaped pattern when WHO cutoffs were applied. It can be seen that different conclusions may appear when evaluating the relationship between obesity and lung function with different classification criteria of obesity degree. For the first time, we systematically assessed the association between visceral fat levels and FEV1%predicted and FVC %predicted using smoothing curve analysis and stratified analysis. We defined the specific range of cutoff points. The possible deviation of the results due to the inconsistency of the visceral fat level's classification criteria was avoided.

Most previous studies have suggested an inverse relationship between obesity and changes in lung function [13, 16]. However, it is essential to note that most studies have only compared lung function in overweight or obese people with average weight [14, 16, 20], ignoring the possible effects of low body fat on lung function. Of course, we know that obesity is a severe problem in the world today. Obesity has reached epidemic proportions, affecting people of all ages and social classes around the world [21, 22]. But the issue of low body fat due to poor nutrition is not trivial either. In addition to the common malnutrition caused by poverty, there are also some phenomena around the world that damage the health caused by the unscientific excessive weight loss due to the incorrect assessment of their own figure and the deviation of their self-perception of obesity [23, 24]. Poor nutrition or low body fat can have several adverse effects on the respiratory system by affecting lung growth, lung structure and function, increasing hospital admissions, and the risk of death in patients with lung disease [25–27]. Our study found that very low and very high VAI levels were closely associated with impaired lung function, showing a significant segmented effect.

So how does visceral fat affect lung function? Since BMI and waist circumference cannot correctly assess body fat distribution, the effect of visceral fat on lung function has attracted increasing attention. The impact of visceral fat on lung function is very complicated, but both chemical and physical mechanisms. First of all, adipose tissue is an essential endocrine organ of the body. When the body’s energy intake exceeds consumption, excessive accumulation of white fat exceeds standard physiological tolerance, resulting in fat overflow, lipid metabolism disorders, insulin resistance, chronic inflammation, metabolic oxidative stress and a series of lipotoxic reactions [28]. Compared with subcutaneous adipose tissue, visceral adipose cells have higher generation and decomposition activity and produce more pro-inflammatory factors, thus impair lung function. Second, excessive visceral fat accumulation can reduce lung compliance and lung volume. And visceral fat can also impair lung function by altering the diaphragm movement, which changes the respiratory system’s mechanics [29, 30]. Finally, as one of the indicators reflecting the body’s nutritional status, the too low visceral fat level may lead to the decline of lung function by impairs the lung’s defence mechanism, affects the control of respiration and the normal contraction of respiratory muscles and induces inflammation.

Conforming with the results of most previous studies on the relationship between obesity and lung function [8, 31–33], we also found that the relationship between visceral adipose tissue and lung function was different between males and females: the VAI level of male participants showed a significant inverted U-shaped relationship with lung function, while the VAI level of female participants showed no significant association with lung function. At present, there is no direct evidence to explain this phenomenon, which may be related to the following factors: First, lung morphology and sex hormone differences in different sexes may affect lung function [34, 35]. Besides, our study found that the level of VAI in males was significantly higher than that in females (13.17 ± 3.91 vs. 7.58 ± 2.65), and the difference was statistically significant. A study has also found that males have a higher area of visceral adipose tissue than females [36]. Therefore, it is reasonable to speculate that the sex difference in the relationship between VAI and lung function may also be related to the difference in fat distribution patterns between males and females.

Inevitably, there are some shortcomings in our study. First, our study type is a cross-sectional design. Because pulmonary function related indicators and VAI were obtained simultaneously, the causal relationship between them could not be determined. Secondly, our study population came from a rural area in Xinjiang, China, a single-centre study, and relevant conclusions still need to be further verified by the external people. Finally, although we tried to analyze the specific relationship between VAI and lung function through stratification study, the stratification factors collected were limited due to the questionnaire's limitations.

## Conclusion

In conclusion, we report for the first time an inverted U-shaped relationship between VAI and lung function. Importantly, we used the curve model to determine the highest inflexion point for VAI among male participants. However, this association was not significant among local female participants. Finally, our study’s conclusions are preliminary and need to be further verified by large-scale, multi-centre prospective follow-up studies.

## Supplementary Information

**Additional file 1:** **Table S1.** Characteristics of 1196 females and 1171 males included in this study.

**Additional file 2:** **Table S2.** Threshold effect analyses of association between VAI levels and lung function FEV1% predicted, FVC% predicted using two-piecewise regression models in female participants.

## Data Availability

Additional material and all other data for this study can be available from the corresponding authors with reasonable request.

## Abbreviations

BMI: Body mass index
VAI: Visceral adiposity index
CKD: Chronic kidney disease
FEV1: Forced expiratory volume in 1 s
FVC: Forced vital capacity
COPD: Chronic obstructive pulmonary disease
