# Supplementary material for: Nonlinear relationship between visceral adiposity index and lung function: a population-based study
Source: Respir Res. 2021 May 24;22:161. doi: 10.1186/s12931-021-01751-7 (PMC8146652; doi:10.1186/s12931-021-01751-7)
Supplement: Supplementary file 1 — Additional file 1: Table S1. Characteristics of 1196 females and 1171 males included in this study. [file 12931_2021_1751_MOESM1_ESM.docx]

**Additional file 1 Table 1 Characteristics of** **1196 females and 1171 males included in this study**

| Variables | | male(n＝1171) | female(n＝1196) | Total(n＝2367) | P-value |
| --- | --- | --- | --- | --- | --- |
| Age, year^Δ^ | | 55.46±9.78 | 51.80±8.77 | 53.61 ± 9.46 | ＜0.001 |
| BMI, kg/m^2Δ^ | | 25.49±3.94 | 25.92±4.62 | 25.71 ± 4.30 | 0.024 |
| VAI^Δ^ | | 13.17±3.91 | 7.58±2.65 | 10.35 ± 4.35 | ＜0.001 |
| Education level^*^ | |  |  |  | 0.217 |
|  | illiterate | 162 (13.83%) | 187 (15.64%) | 349 (14.74%) |  |
|  | Primary and so on | 1009 (86.17%) | 1009 (84.36%) | 2018 (85.26%) |  |
| Gross annual income, yuan^*^ | |  |  |  | ＜0.001 |
|  | ＜10000 | 575 (49.10%) | 724 (60.54%) | 1299 (54.88%) |  |
|  | ≥10000 | 596 (50.90%) | 472 (39.46%) | 1068 (45.12%) | ＜0.001 |
| Active smoking^*^ | |  |  |  |  |
|  | Yes | 308 (26.30%) | 8 (0.67%) | 316 (13.35%) |  |
|  | No | 863 (73.70%) | 1188 (99.33%) | 2051 (86.65%) |  |
| Passive smoking^*^ | |  |  |  | ＜0.001 |
|  | Yes | 119 (10.16%) | 42 (3.51%) | 161 (6.80%) |  |
|  | No | 1052 (89.84%) | 1154 (96.49%) | 2206 (93.20%) |  |
| Chronic bronchitis^*^ | |  |  |  | ＜0.001 |
|  | Yes | 172 (14.69%) | 252 (21.07%) | 424 (17.91%) |  |
|  | No | 999 (85.31%) | 944 (78.93%) | 1943 (82.09%) |  |
| COPD^*^ | |  |  |  | 0.028 |
|  | Yes | 161 (13.75%) | 129 (10.79%) | 290 (12.25%) |  |
|  | No | 1010 (86.25%) | 1067 (89.21%) | 2077 (87.75%) |  |
| Hypertension^*^ | |  |  |  | 0.879 |
|  | Yes | 313 (26.73%) | 323 (27.01%) | 636 (26.87%) |  |
|  | No | 858 (73.27%) | 873 (72.99%) | 1731 (73.13%) |  |
| Peptic ulcer^*^ | |  |  |  | 0.186 |
|  | Yes | 81 (6.92%) | 100 (8.36%) | 181 (7.65%) |  |
|  | No | 1090 (93.08%) | 1096 (91.64%) | 2186 (92.35%) |  |
| Cholecystitis^*^ | |  |  |  | ＜0.001 |
|  | Yes | 182 (15.54%) | 343 (28.68%) | 525 (22.18%) |  |
|  | No | 989 (84.46%) | 853 (71.32%) | 1842 (77.82%) |  |
| CKD^*^ | |  |  |  | 0.003 |
|  | Yes | 104 (8.88%) | 68 (5.69%) | 172 (7.27%) |  |
|  | No | 1067 (91.12%) | 1128 (94.31%) | 2195 (92.73%) |  |
| Osteoporosis^*^ | |  |  |  | ＜0.001 |
|  | Yes | 50 (4.27%) | 108 (9.03%) | 158 (6.68%) |  |
|  | No | 1121 (95.73%) | 1088 (90.97%) | 2209 (93.32%) |  |
| [Fracture](javascript:;)^*^ | |  |  |  | 0.002 |
|  | Yes | 95 (8.11%) | 59 (4.93%) | 154 (6.51%) |  |
|  | No | 1076 (91.89%) | 1137 (95.07%) | 2213 (93.49%) |  |
| FVC% predicted^Δ^ | | 95.63±19.80 | 97.47±19.38 | 96.54 ± 19.61 | 0.024 |
| FEV1%predicted^Δ^ | | 86.79±20.65 | 89.23±20.18 | 88.00 ± 20.45 | 0.004 |

^*^Categorical variables are presented as frequency and percentage, n (%), using Pearson chi-square test to compare differences. ^Δ^Continuous variables are presented as mean value ± standard deviation, and Student's t-test is used to compare the differences between each group.

*BMI* body mass index, *VAI* visceral adiposity index, *CKD* chronic kidney disease, *FEV1* forced expiratory volume in 1 s, *FVC* forced vital capacity
