# Supplementary material for: Nonlinear relationship between visceral adiposity index and lung function: a population-based study
Source: Respir Res. 2021 May 24;22:161. doi: 10.1186/s12931-021-01751-7 (PMC8146652; doi:10.1186/s12931-021-01751-7)
Supplement: Supplementary file 2 — Additional file 2: Table S2. Threshold effect analyses of association between VAI levels and lung function FEV1% predicted, FVC% predicted using two-piecewise regression models in female participants. [file 12931_2021_1751_MOESM2_ESM.docx]

**Additional file 2 Table 2** **Threshold effect analyses of association between VAI levels and lung function FEV1% predicted, FVC% predicted using two-piecewise regression models in female participants**

| VAI | | ^*^Unadjusted |  |  | VAI | ^Δ^Adjusted |  |
| --- | --- | --- | --- | --- | --- | --- | --- |
|  |  | β(95%CI) | P-value |  |  | β(95%CI) | P-value |
| FEV1% predicted | |  |  |  |  |  |  |
|  | ＜15.2 | 0.47 (-0.01, 0.94) | 0.057 |  | ＜15.0 | 0.48 (-0.01, 0.97) | 0.056 |
|  | ≥15.2 | -1.26 (-3.67, 1.15) | 0.305 |  | ≥15.0 | -0.97 (-3.03, 1.09) | 0.354 |
| Likelihood ratio test p | |  | 0.187 |  |  |  | 0.203 |
| FVC% predicted | |  |  |  |  |  |  |
|  | ＜15.3 | 0.01 (-0.46, 0.47) | 0.981 |  | ＜14.8 | 0.01 (-0.44, 0.46) | 0.971 |
|  | ≥15.3 | -1.90 (-4.32, 0.52) | 0.124 |  | ≥14.8 | -2.60 (-5.49, 0.29) | 0.078 |
| Likelihood ratio test p | |  | 0.091 |  |  |  | 0.145 |

*VAI* visceral adiposity index, *FEV1* forced expiratory volume in 1 s, *FVC* forced vital capacity

^*^No variables have been adjusted

^Δ^Adjusted for Age, economic income, education level, smoke exposure and other vital covariates including previous chronic bronchitis, COPD, hypertension
